# Corruption in the health sector: A problem in need of a systems-thinking approach

**DOI:** 10.3389/fpubh.2022.910073

**Published:** 2022-08-24

**Authors:** Emily H. Glynn

**Affiliations:** Department of Laboratory Medicine and Pathology, University of Washington, Seattle, WA, United States

**Keywords:** corruption, global health, health systems strengthening, systems-thinking approach, low- and middle- income countries (LMICs)

## Abstract

Health systems are comprised of complex interactions between multiple different actors with differential knowledge and understanding of the subject and system. It is exactly this complexity that makes it particularly vulnerable to corruption, which has a deleterious impact on the functioning of health systems and the health of populations. Consequently, reducing corruption in the health sector is imperative to strengthening health systems and advancing health equity, particularly in low- and middle-income countries (LMICs). Although health sector corruption is a global problem, there are key differences in the forms of and motivations underlying corruption in health systems in LMICs and high-income countries (HICs). Recognizing these differences and understanding the underlying system structures that enable corruption are essential to developing anti-corruption interventions. Consequently, health sector corruption is a problem in need of a systems-thinking approach. Anti-corruption strategies that are devised without this understanding of the system may have unintended consequences that waste limited resources, exacerbate corruption, and/or further weaken health systems. A systems-thinking approach is important to developing and successfully implementing corruption mitigation strategies that result in sustainable improvements in health systems and consequently, the health of populations.

## Introduction

The health sector is a dynamic system composed of complex interactions between patients, providers, payers, suppliers, and policy makers. It is exactly this complexity that makes it particularly vulnerable to corruption. Corruption, commonly defined as the “abuse of entrusted power for private gain,” (1) is a problem within health care systems globally. However, it is important to note that “corruption” not only encompasses actions that are illegal in most countries, but also those that could reasonably be considered unethical, and when pervasive, weaken and foster distrust in the health systems.

Corruption takes many forms within the health sector and occurs at all organizational levels from government agencies to the direct provision of care. Likewise, the motivations underlying health sector corruption vary by country. Therefore, it may be challenging to adapt corruption-mitigating strategies that were successful in one health system to another system with completely different incentives, accountability structures, enforcement mechanisms, and socio-economic and political contexts. Given the heterogeneity and dynamic nature of health systems, sustainable reductions in corruption and resultant improvements in health care delivery require a systems thinking approach.

In order to understand the scope of corruption, its impact on population health and health systems will be reviewed. This will be followed by an overview of common types of health sector corruption with special attention paid to differences in manifestations of and motivation and policies underlying corruption in high-income countries (HICs) and low- and low-middle-income countries (LMICs). The second section will review select anti-corruption strategies that have been implemented in LMICs through systems-thinking lens and how a systems-thinking approach could be utilized to address health sector corruption, particularly in LMICs.

## Impact of corruption on population health and health systems

Pervasive corruption has the potential to impact the health of populations. Countries with high levels of corruption spend less on health care as a percentage of gross domestic product (2, 3). In addition, high levels of corruption correlate with poor health-related outcomes. This includes higher infant and child mortality rates (4, 5), lower life expectancy (2, 5), lower immunization rates (6), and higher rates of antibiotic resistance (7). Moreover, corruption has a negative effect on the mental health of citizens, with individuals who experience high levels of corruption reporting a lower perception of their overall health (8, 9).

Corruption impacts health systems as well. In 2019, the U.S. government recovered $3.6 billion USD in health-related fraud judgements and settlements (10). However, this likely represents the tip of the iceberg of fraudulent activities in U.S health system, which is estimated to lose $58.5–83.9 billion USD annually to fraud and abuse (11). This trend is also reflected in global estimates of health care spending, where at least 7% is ceded to corruption, an estimated $500 billion USD (12). These data suggest that commitment of financial resources may have a diminished impact on the health of populations if they are being diverted for corrupt purposes.

Lastly, corruption is particularly problematic because of who is most affected. Previous studies have shown that corruption impacts the most vulnerable patients regardless of country. Individuals who are in poor health (13) or are at high socioeconomic risk (3, 14) are more likely to make informal payments. Data from sub-Saharan Africa suggests that individuals who reported paying bribes for health-related services were 4 to 9 times more likely to also report difficulty accessing health care (15). In the United States, nearly 790,000 Medicare beneficiaries over a 3-year period were treated by providers who were subsequently found to have committed fraud and abuse violations (16, 17). These beneficiaries were more likely to be non-white, dually eligible for Medicare and Medicaid (suggesting lower income), and disabled (16).

These examples highlight the deleterious impact of corruption on population health, health systems, and addressing health equity. Consequently, tackling corruption within the health sector is imperative to strengthening health systems. Understanding the forms of health sector corruption is an important first step in these mitigation efforts.

## Manifestations of corruption in health systems

**Figure 1:**
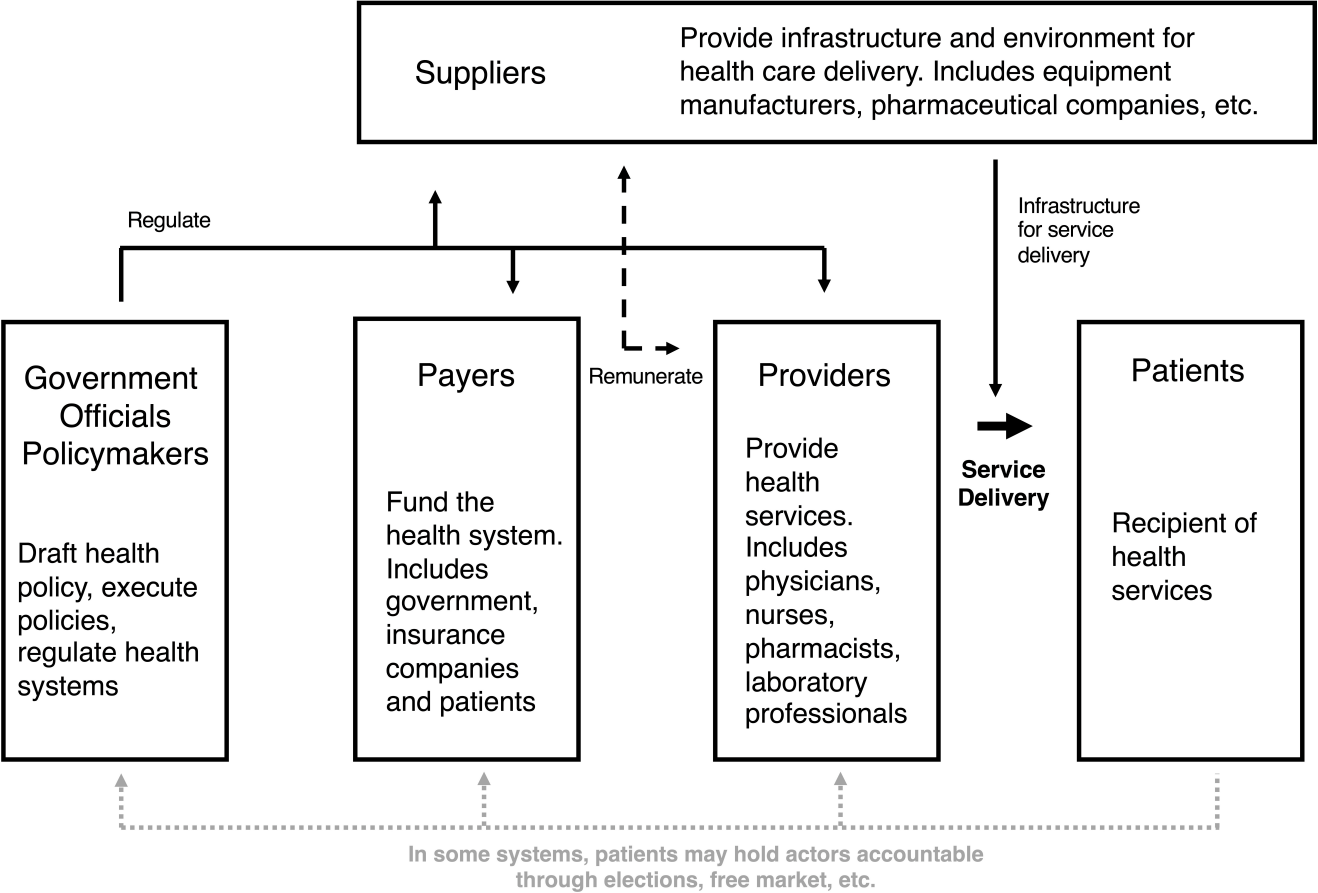
Schematic diagram of actors in the health system along the continuum of service delivery.

In order to understand manifestations of health sector corruption, it is important to be familiar with actors in health systems and their relationships to one another. The exact actors vary from country to country, but roles within health systems can be characterized based on a continuum of service delivery (Figure 1). On one spectrum of health systems, furthest removed from direct provision of services, are governments and the government officials who are responsible for crafting health-related policies, executing the policies, and regulating the health system. At the level of direct service delivery are the health care workers who provide services (e.g., physicians, nurses, pharmacists, etc.), and patients who are the recipients of those services. In between the actors involved in policy and regulation and those involved in the direct provision of care are the payers and suppliers. Payers fund the health system and, depending on the country, may be government agencies, non-profit or for-profit insurance companies, or patients themselves. Suppliers are those that provide the infrastructure and environment for health care to be delivered, e.g., medical device and pharmaceutical companies, equipment manufacturers, etc.) (18). Importantly, corruption can occur at any level and involve any actor within this complex system. The six forms of health sector corruption reviewed in detail here are improper financial relationships, theft and diversion of resources, fraudulent billing, absenteeism, informal payments, and counterfeit medical supplies (summarized in Table 1).

**Table 1:** Forms of health sector corruption and the actors who are involved.

**Corruption type**	**Definition**	**Actors involved**	**Examples**
Improper financial relationships	Relationships between actors within the health system that have the potential to create situations where individuals are motivated by financial enrichment over medical indication, patient well-being, and/or public health	Government officials Payers Suppliers Providers	Provider who receives financial support from pharmaceutical companies that manufacture medications that the provider prescribes to patients at their clinic
Fraudulent billing and claims	Obtaining reimbursement for services or items that were either not provided, more complex than what was provided, or medically unnecessary	Providers Suppliers	Upcoding, seeking reimbursement for a procedure that was not actually performed, unbundling diagnostic testing to increase reimbursement
Theft and diversion	Theft - taking resources to which one is not entitled without consent or permission. Diversion - taking and reselling resources for another purpose without consent or permission	Government officials Payers Suppliers Providers	Taking supplies from a public hospital for use in one's private clinic, diverting medications for resale
Absenteeism	Frequent, unauthorized absences for the purpose of pursuing private business during working hours	Government officials Providers	Taking frequent absences from public sector health center to work in a private clinic
Informal payments	“Payments to individual and institutional providers, in kind or in cash, that are made outside of official payment channels or are purchases meant to be covered by the health care system”	Government officials Payers Suppliers Providers	Soliciting or offering a bribe or gift to shorten patient wait times at a busy clinic, charging more than an official user fee and pocketing the difference
Counterfeit medical supplies	Intentional production and distribution of falsified medical supplies for financial gain	Government officials Suppliers	Bribing government officials to waive required inspections allowing the import of counterfeit diagnostic test kits; selling antibiotics with no active ingredient to patients who cannot afford to pay for the authenticated version

### Improper financial relationships

Improper financial relationships are associations between actors within the health system that have the potential to create a conflict of interest. Specifically, they foster situations where individuals are motivated by financial enrichment over medical indication, patient well-being, and/or public health. At the highest level of service delivery, improper financial relationships can occur between government officials and for-profit entities within the health sector (e.g., pharmaceutical, medical device, insurance companies) (19). Other potential manifestations of improper relationships at the highest level of government include deregulation of the health sector to the benefit specific interest groups, influence over health-related recommendations or guidelines, expediting approval of pharmaceuticals or medical devices, etc. (18, 20).

Improper financial relationships involving providers can also exert inappropriate influence at the level of direct service delivery. Two common business relationships that fall within this category are self-referrals and kickbacks. Self-referrals occur when providers refer patients for medical services performed by an entity with whom the provider or family member has a financial relationship. Although they may be legal, these financial relationships have the potential to result in medically unnecessary interventions or more expensive interventions that financially enrich providers at the expense of patients or payers (21). Kickbacks at the service-delivery level are similar to those at the government or payer level. For example, a pharmaceutical company may pay inducements to providers to preferentially prescribe their company's medication (22).

### Fraudulent billing and claims

Fraudulent billing refers to the act of obtaining reimbursement for services or items that were either not provided, more complex than what was provided, or medically unnecessary. The actors involved in fraudulent billing can vary depending on how health care was financed. In countries with social health insurance programs, fraudulent billing occurs primarily between providers and either government or private payers. In countries without well-established health insurance systems where out-of-pocket payments predominate, providers may fraudulently obtain reimbursement from patients. In addition, providers may also defraud the government for services or items related to certain diagnoses, patient populations, or conditions that are provided by government at no charge to patients (HIV, tuberculosis, prenatal or pediatric care). Fraudulent billing is a relatively common form of health sector corruption in HICs. In OECD countries, fraudulent billing in the form of overprovision or overbilling for services were among the most common forms of corruption (20, 23, 24).

### Theft and diversion

Theft occurs when individuals take resources to which they not entitled without consent or permission. Diversion refers to taking and reselling resources for another purpose without consent or permission. Theft and diversion of resources can occur at all levels of a health system. At the government or payer level, theft often takes the form of embezzlement, where government officials or insurance company employees siphon health-related funding for personal use (20). In addition, large-scale theft of donor funding allocated to LMICs by government officials has also been reported (25).

At the provider level, health care workers may divert supplies, medication, equipment, or official fees for financial enrichment (26–29). The extent of theft and diversion at the provider level is challenging to precisely measure. Relative to other forms of corruption, theft and diversion is perceived to be less common in OECD countries (20). However, qualitative studies from sub-Saharan Africa, indicate that theft may be a larger concern in this region where public health systems have historically been weak (26, 27). Health care workers from multiple sub-Saharan African countries report having personal experience with theft within the health system (26–29) and cite low public-sector salaries and suboptimal working conditions as reasons for theft and diversion (26, 27).

### Absenteeism

Frequent, unauthorized absenteeism is regarded as corrupt when public sector workers “choose to engage in private pursuits during working hours” (12). Although absenteeism can occur at the highest levels of government, this review will focus on absenteeism of health care workers and its impact on the direct provision of care. Commonly cited factors driving absenteeism include low and/or unreliable salaries in the public sector, lack of monitoring and accountability, and substandard work environments that includes demanding workloads partially induced by frequent absenteeism (27, 28, 30–38). Specifically, low and/or unreliable salaries are a major driver of absenteeism. Qualitative studies of absenteeism among public sector health care workers in sub-Saharan Africa illustrate the challenges these individuals face. In Nigeria, public sector health care workers report being unable to cover basic necessities with their salaries, including food, clothing, transportation, etc. (39). Some of these employees report going 1 year without being paid a salary (39).

Poor and/or intermittent remuneration promote absenteeism when health care workers engage in dual-practice, or the provision of clinical care in the public and private sector concurrently (40). Although dual-practice occurs in countries at all income levels (40), it is particularly problematic for service delivery when health care workers are absent from their public sector position in order to provide care in the private sector (27, 39, 40). In many HICs where governance is stronger, the private sector is formalized, and the health systems are well-developed, dual practice is prohibited or well-regulated and therefore less likely to result in absenteeism (40). However, many LMICs have weaker governance structures and health systems resulting in a blurred separation of the public and private sector and weak or non-existent regulation of the private sector. These factors contribute to poor regulation of dual practice and incentivizes absenteeism (40).

### Informal payments

Informal payments are defined as “payments to individual and institutional providers, in kind or in cash, that are made outside of official payment channels or are purchases meant to be covered by the health care system” (41). They can involve actors at all levels of the health care system from government officials, suppliers, and providers. Informal payments can be illegal or legal and encompass a broad range of unofficial exchanges including overt bribes, favors, substantial gifts, and payments solicited under the guise of an official transaction or fee (42). Some of the motivations underlying informal payments are similar to those described for absenteeism and theft/diversion, namely, low public health salaries (43–46). In addition, cultural and societal norms around gift-giving (44, 46), the marketization of health care (44–46), and prevalence of bribery in other sectors of society (37) are also cited as reasons for informal payments.

### Counterfeit medical supplies

Lastly, counterfeit therapeutics, medical devices, and other medical supplies represent an important form of corruption that disproportionately impacts health systems in LMICs (47). According to a report by the World Health Organization (WHO), 20% of malaria medications, 17% of antibiotics, and 9% of anesthetics/analgesics circulated globally were either substandard or falsified (47). Although these substandard or falsified products were reported in numerous countries of all income levels, the problem is particularly acute in Africa, which represented 42% of the total reports (47). Another study evaluating medications in Latin America identified a negative correlation between the quality medications and the level of corruption within the country (48). It is important to note that while producing and distributing intentionally falsified supplies represents a form of corruption, substandard products may be a result of technical inexperience or weak capacity.

Potential factors giving rise to the circulation of counterfeit medical supplies include poor governance in many LMICs where the regulatory capacity is inadequate to ensure the authenticity of these products (47). This regulation is further complicated by the fact that many of these supplies are the product of complex multinational supply chains. Regulation may be even more challenging in LMICs without a national insurance program and where patients are paying for these supplies out-of-pocket. Moreover, those who are suspicious of the efficacy of the medication or device may be reluctant to voice their concerns out of fear of reprisal from criminal enterprises involved in trafficking (47). As highlighted by these examples, while counterfeit medical products occur in countries of all income level, the reporting available suggests the impact is felt most by patients in LMICs.

### Corruption in LMICs vs. HICs

The above examples demonstrate that health sector corruption is a global problem with a heterogeneous presentation. For example, fraudulent billing is particularly problematic in countries with some form of social health insurance. In contrast, while theft/diversion, informal payments, absenteeism, and counterfeit medications are present in the health systems of many LMICs, they are less common in HICs. These distinctions highlight the structural differences between health systems in LMICs and HICs, including differing incentives, regulations, policies, forms of remuneration, resources, etc. Moreover, this heterogeneity underscores the need for a systems-thinking approach to address corruption the health sector.

Although corruption occurs in countries of all income levels, this review will focus on using a systems-thinking approach to understand corruption within the health sector in LMICs for two main reasons. The first is that the majority of the most corrupt countries according to Transparency International's Corruption Perceptions Index (CPI) (49) are categorized as low-income or low-middle income (50). The second and more relevant reason is that corruption represents an informal institution in many LMICs (51). As with most institutions, corruption becomes self-reinforcing, fostering an equilibrium of continued corruption that is challenging to disrupt (51). For this reason, using reductionist strategies to address corruption within health systems of LMICs is unlikely to result in sustainable improvement and may even further exacerbate the problem.

## Applying a systems lens to health sector corruption: Structures beneath the surface

**Figure 2:**
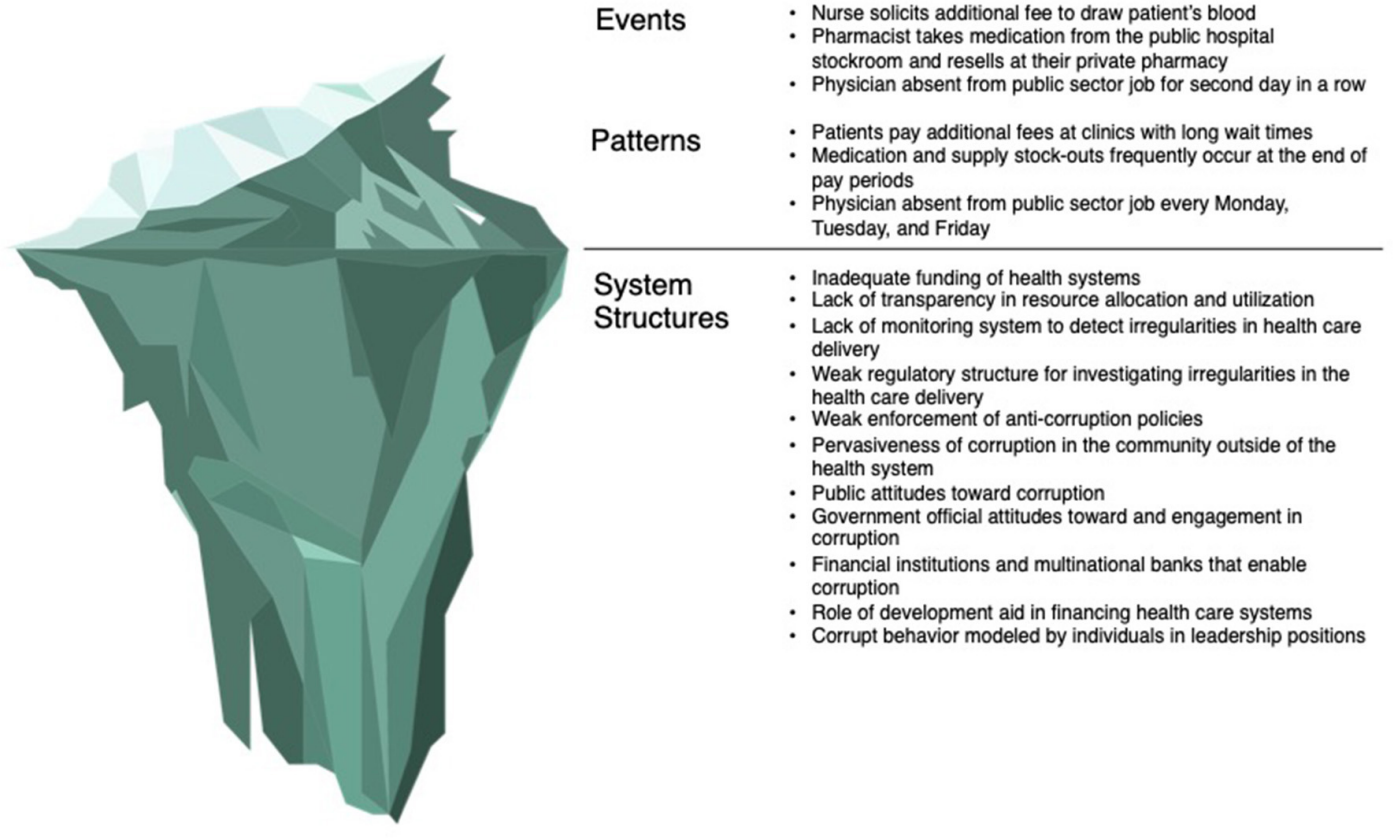
Iceberg diagram of health sector corruption.

The above forms of health sector corruption represent the tip of the iceberg, the events and patterns that are readily visible to observers. However, effectively and sustainably reducing corruption requires an understanding of what is underneath the surface – the structure of health systems, the political and socio-economic environment, and historical context that drive these visible manifestations of corruption (52). This section will summarize the environmental factors that enable and perpetuate corruption within health systems (Figure 2), with special attention paid to differences in corruption within LMICs and HICs.

### Socio-economic factors

Although corruption occurs in health sectors of countries at all stages of economic development, the underlying motivations often differ between HICs and LMICs. As outlined in the previous section, absenteeism, informal payments, theft and diversion, and counterfeit medical supplies are forms of health sector corruption that appear to be particularly problematic in LMICs. When evaluating the determinants of these forms of corruption, recurrent patterns that emerge include low and/or unreliable salaries for health care workers and substandard working conditions in the public sector (22, 28, 30, 32, 34, 38, 43–45). When these factors combine with minimal oversight, corrupt individuals in positions of leadership, and corruption in other areas of society (22, 37, 38, 53) it is unsurprising that corruption represents an institutional reality for health care workers in LMICs rather than a scheme for personal enrichment as is seen in many HICs (54). These differences in motivations require a different framework for thinking about corruption in LMICs in order to develop effective mitigation strategies.

To explore these important differences in motivating factors that inform the type and scope of corruption, Monika Bauhr puts forth a framework of “need” vs. “greed” corruption (54). “Need” corruption refers to acts of corruption that are necessary to carry out in order to access services to which citizens are legally entitled. For example, patients are compelled to make informal payments in order to access health care services that should be provided at no or reduced cost by the government. Health care workers have limited choices but to engage in dual practice or to divert supplies or medications in order to supplement unsustainable public sector salaries. In contrast, “greed” corruption refers to acts of corruption that are carried out by actors for the purpose of personal advantage (54). Embezzlement of health care funds at the government or payer level and some forms of fraudulent billing or improper financial relationships are arguably examples of “greed” corruption.

While greed-based corruption occurs in countries regardless of income level, need-based corruption is relatively uncommon in HICs (54). Moreover, Bauhr suggests that need-based corruption is associated with lower trust in institutions, an observation that was not seen with greed-based corruption (54). Given these differences in the trust of institutions and governments, mitigating need-based and greed-based corruption will require different strategies. There is no doubt that corruption occurs in HICs and may even result in larger financial losses. However, in many HICs, there is an institutional and legal framework for investigating corrupt actors and holding them accountable as well as trust among citizens that this will occur. Understanding these motivators is critical to a systems-thinking approach to reduce corruption in the health sector. Although these institutions may exist outside of the health system and well-beneath surface of the metaphorical iceberg, any anti-corruption strategy must understand the institutional context as they influence the personal and work environments of actors within the health system.

### Health systems

Another factor beneath the surface of the health sector corruption iceberg is the strength of health systems in LMICs. A significant barrier to improving health outcomes in LMICs are weak health systems (55). One potential explanation for these weak systems is the wave of structural adjustment programs (SAPs) that were imposed on low-income countries (LICs) by international financial institutions starting in the 1980's (56). These neoliberal policies required heavily indebted LICs, particularly in sub-Saharan Africa, to reduce public sector spending and enhance privatization and deregulation in exchange for debt reduction (57). Some have argued that policies enacted in the health sector to comply with SAPs destabilized public health systems; these policies include cuts to public health resources and/or diversion of resources to the private sector, institution of user fees to access health services, and lay-offs or salary reductions of public sector health care workers (56).

Neoliberal policies represent potential explanation for the weak public health systems that are pervasive in LMICs. These weak systems fail to deliver services to the public and create an environment where the consequences of not engaging in corruption outweigh any potential benefits to holding corrupt actors accountable (58). This relationship between health sector corruption and weakened health systems is essential to addressing corruption in LMICs and may help to explain why anti-corruption strategies developed in HICs may fail to deliver in LMICs. They also highlight how anti-corruption strategies without concomitant investments in strengthening the health sector, may do little to reduce health sector corruption.

### Donors and development aid

When considering how to address health sector corruption in LMICs, it is not only important to understand the context of the health system, but also the socio-economic and political environment in which these health systems exist. One important distinction between the environment within LMICs and HICs, particularly when considering financing of health systems, is the role of donors and development aid. From 1990 to 2014, nearly $460 billion USD in development aid was disbursed from high-income to developing countries (59). Donor funding is estimated to represent 30% of health care expenditures in low-income countries (LICs) (12). This proportion is even higher for HIV-, malaria-, and tuberculosis-related care where donor funding of these disease entities is over two times the amount spent by ministries of health (12).

Although investments in the health sector made possible through development aid has saved countless lives, it is important to understand the role of donors within health systems and health sector corruption as development aid continues to be allocated to corrupt countries (60, 61). In sub-Saharan Africa specifically, aid as a percentage of GDP and government expenditure are negatively correlated with quality of governance, even after controlling for GDP per capita (62). Specific to the health sector, approximately $34 million USD of development aid was diverted from the Global Fund (25), leading to significant changes in policies related to transparency and accountability (63). However, it remains to be seen whether these strategies are effective in addressing corruption (63). Therefore, the presence of donors and donor funding adds another layer of complexity to health systems in LMICs. Systems thinking can be utilized to better understand the role of development aid and its interactions with other variables that contribute to health sector corruption.

## Applying a systems lens to health sector corruption: Effectiveness of anti-corruption strategies

The evidence indicates that corruption is problem that must be addressed to strengthen the health systems of LMICs. Goals of modern anticorruption strategies include strengthening accountability, detection, and enforcement; improving transparency; and preventing corruption through provision of resources. Examples of strategies utilized to achieve each of these goals are outlined in Table 2. Unfortunately, there is a dearth of strong evidence supporting the efficacy of anti-corruption reforms in the health sector and strongest evidence was for programs implemented in HICs (64). Given the significant differences between health systems in HICs and LMICs highlighted above, it is unclear whether these strategies can be adapted in other settings with the same success. Moreover, many anti-corruption strategies address individual interactions or behaviors, but do not explore how those interactions fit within the context of the system. This section summarizes the effectiveness of three strategies that have been utilized in LMICs to reduce corruption: anti-corruption agencies to strengthen accountability and enforcement, community engagement to improve transparency, and raising public sector salaries to prevent corrupt behavior through provision of resources. These strategies will be reviewed in a systems-thinking context to highlight the limitations of viewing corruption within the health system as isolated linear relationships.

**Table 2:** Examples anti-corruption theories and corresponding strategies.

**Anti-corruption theory**	**Example strategies**
Strengthening accountability, detection, and enforcement	• Anti-corruption agencies • Improving technical infrastructure to detect irregularities • Legal framework for prosecution of health sector corruption
Increasing transparency	• Community monitoring boards • Anti-corruption media campaigns • Publicizing performance metrics for health care worker and facilities (i.e. report cards) • Publicizing resource allocation and spending in health • Disclosure of financial relationships
Prevention	• Increasing health care worker salaries • Allocated resources to the health sector to improve working conditions • Incentives for “clean behavior”

### Anti-corruption agencies

In the systematic review cited above, the study that provided the strongest indication of success was a series of legislative and executive efforts in the U.S. aimed at curbing fraud and abuse in Medicare and Medicaid (64). These efforts included formation of an anti-corruption task force with prosecutorial authority and upgrading the analytic capacity for improved detection of billing irregularities (64). As a result of increased detection of fraudulent activities and resultant convictions, the anti-corruption task force was estimated to have recovered $1–3 billion USD per year over the course of 10 years (64).

Formation of independent anti-corruption agencies has also been attempted in LMICs, but with mixed results. For example, in Karnatka, India, an anti-corruption agency underwent a change in scope and leadership in 2001 to address rampant public sector corruption. Under new leadership, this agency uncovered systemic corruption within the health sector partly through an increase in citizen reporting. However, there was no concomitant increase in convictions for corrupt acts as a result of this improved detection. One reason for this lack of enforcement was the weak political support for this agency's activities, limiting its ability to investigate and prosecute the corrupt behavior it uncovered, particularly at higher levels of the government (65).

In contrast to the experience in Karnatka, an anti-corruption agency in Uganda was granted substantial enforcement authority and was formed by the president himself in response to pervasive health sector corruption (66). This agency was responsible for a significant decline in bribery among health care workers, the recovery of millions in USD worth of stolen health supplies, and the conviction of health care workers for corruption-related crimes. However, without a simultaneous effort to raise salaries and improve working conditions, health care worker morale deteriorated under the agencies aggressive tactics resulting in a prolonged strike that that debilitated the nation's health system (66).

**Figure 3:**
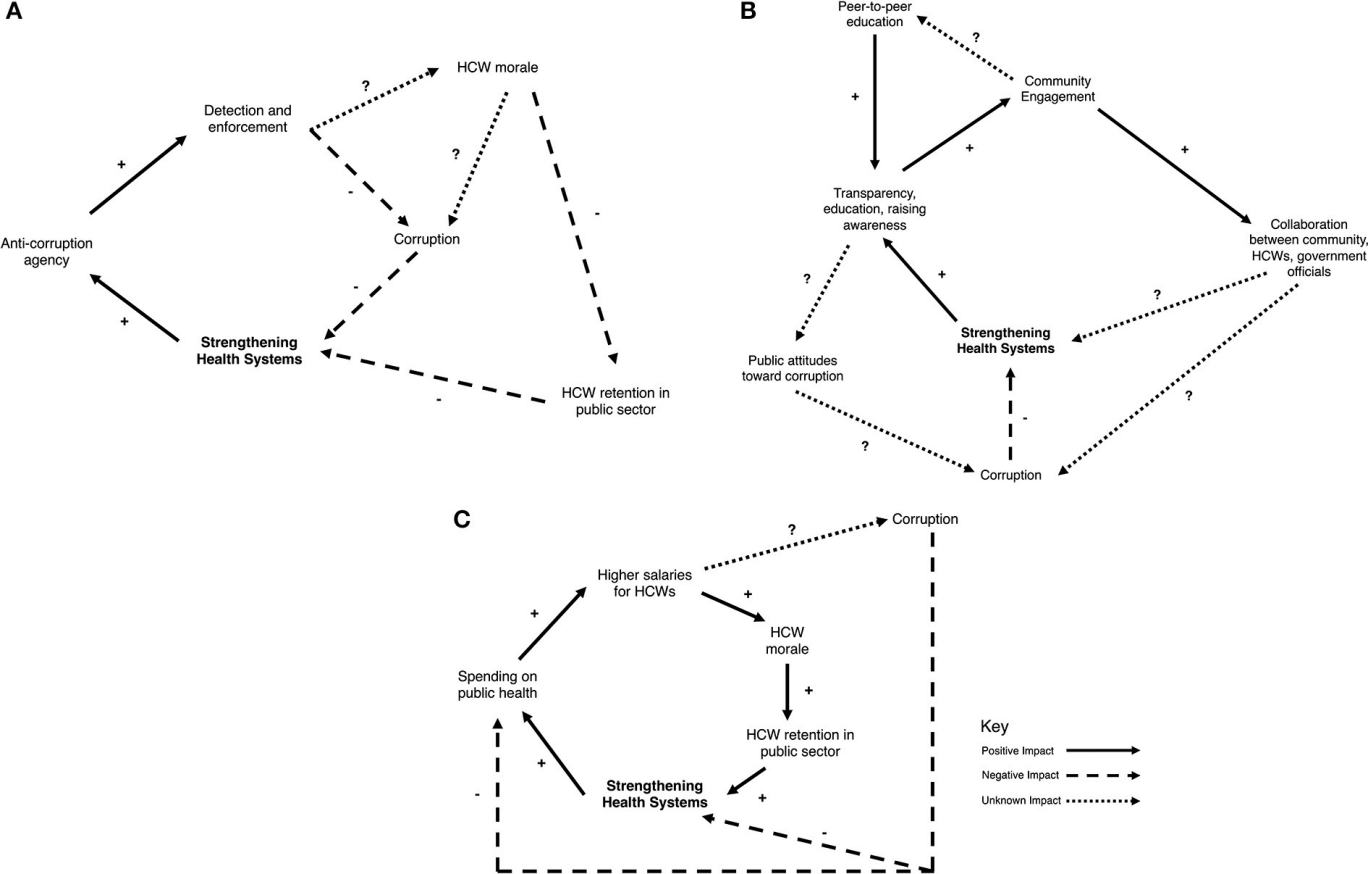
Causal loop diagrams for the following anti-corruption strategies. **(A)** Anti-corruption agency, **(B)** community engagement, and **(C)** raising salaries.

These examples highlight the danger of applying a reductionist, rather than a systems-thinking approach. Forming an anti-corruption agency addresses a component of the system – individual acts of corruption among service providers. However, they do little to address the working conditions, institutional and economic factors, and social norms that enable individuals to ask for a bribe or divert medical supplies. At a minimum, the status quo remains in effect if there is no political backing of the agency or ability to enforce anti-corruption regulation, as highlighted by the example in Karnatka. At their worst, they can result in significant unintended consequences that further weaken the health system, as highlighted by the example in Uganda. Although allocating resources to enhance detection and enforcement has the potential to reduce individual corrupt actions in the short-term, these tactics may only represent a “quick fix.” Over time, aggressive enforcement of corruption in isolation can decrease health care worker morale resulting in increased number of health care workers leaving the public sector. This would have the unintended and delayed consequence of further weakening the health system (Figure 3A).

### Community engagement

Another strategy used to reduce corruption is mobilizing community members to hold actors in the health system accountable through enhanced transparency. For example, the presence of a monitoring board composed of community members in Bolivia was associated with a decrease in informal payments and overpricing for supplies and medications (67). A randomized control trial in Uganda demonstrated that health care service delivery and population health indicators improved when citizens were provided performance metrics on their health facilities and encouraged to engage with health care workers to develop a shared action plan to improve local health outcomes (68). Lastly, formalized citizen feedback can catalyze and inform anti-corruption efforts. Information from social audit surveys that polled perceptions of and experiences with corruption in Nicaragua were used to lobby for anti-corruption policies and ethics training for public officials (69).

Similar to anti-corruption agencies, it is unclear if community engagement as an isolated strategy is sufficient to curb entrenched health sector corruption. For instance, a randomized trial evaluating the effectiveness of a community-based transparency campaign in Tanzania and Indonesia failed to improve health outcomes in the intervention communities (70). In this study, citizens were invited to attend meetings with a facilitator to discuss their experiences with and develop a set of activities to address maternal and newborn health in their community. However, there were no resources or support provided by the program outside of these facilitated meetings. At the conclusion of the study, there was no significant improvement in the use of perinatal and postnatal services, birth weight, or feelings of civic engagement between the intervention and control groups. The authors speculate that it was challenging for participants to operationalize the ideas developed during the facilitated discussions into actions that would lead to tangible improvements (70).

In fact, methods commonly used to engage community members through increased transparency could have the unintended consequence of leading to more corruption. At least two studies have shown that exposing citizens to anti-corruption media actually increased their willingness to pay a bribe (71, 72). The content of the media varied in each study, but included messaging on the pervasiveness of corruption (72), recent corruption scandals, the impact of corruption on communities, and recent anti-corruption efforts undertaken by the government (71). It is possible that anti-corruption media campaigns may perpetuate feelings futility and powerlessness among community members, rather than mobilizing them to combat corruption (71).

These examples address one component of the system – public awareness of corruption. The long-term goal of these awareness building campaigns is to hold those in position of power accountable. However, if enhanced transparency is not coupled with legitimate and visible efforts by health care workers to improve services or government to commit resources to improve the health system or deliver on anti-corruption policies, then advertising the extent of corruption may only perpetuate the perception that corruption is pervasive and inevitable (Figure 3B). This can create a reinforcing loop where citizens believe that corruption is ubiquitous and therefore they engage in corruption. The ultimate result is even more corruption that becomes increasingly institutionalized within the system.

### Raising salaries

Lastly, investing resources in health systems of LMICs, specifically to improve wages of health care workers in the public sector, may itself represent an anti-corruption strategy. Despite increased spending on health care globally over the past 2 decades, there are significant disparities in per capita spending between in HICs ($5,252 USD) and LMICs ($40–81 USD) (73). This disparity in funding may underly the aforementioned pattern seen in LMICs of health care workers engaging in corruption to supplement unsustainably low public sector salaries. Consequently, it is plausible that health care workers may be less likely to engage in dual practice, solicit informal payments, and/or divert supplies and medications to supplement their income if they are paid an sufficient and reliable salary. Adequate investments in health sector infrastructure, equipment, and guarantee of supply chains for therapeutics and consumable supplies can improve access to services, which could also deter perpetuation of an unregulated private sector within health systems of LMICs (74).

Ecological studies incorporating data from numerous countries across multiple continents indicates that, specifically in LMICs, there is an association between higher civil servant salaries and lower corruption (75, 76). However, based on modeling from one of these studies, salaries would need to be increased substantially to eliminate corruption if raising wages was the only strategy used (i.e., in the absence of concomitant enforcement mechanisms to deter corruption) (76). Moreover, on an individual country level, the suggestion that higher salaries alone will reduce corruption is less clear. In 2010, the Ghanaian government doubled police officer salaries, in part to reduce corruption within the police force. However, efforts to solicit bribes and the monetary value of bribes paid to police officers actually increased after 2010, suggesting the higher salaries exacerbated corruption (77). The authors offered potential reasons for this unexpected result. First, raising salaries may have contributed to a sense of entitlement among police officer to expect higher bribes. Also, the higher income may have created additional pressures to financially support extended family members that necessitated the solicitation of more bribes (77).

Although not specific to the health sector, this example highlight the complicated nature of corruption. Supplementing low salaries may be one reason for engaging in corruption, but there are important social and institutional factors that also contributed to a police officer's willingness to solicit a bribe. These other factors may not be readily apparent without utilizing a systems-thinking approach. In this example, raising wages without interventions that address other aspects of the system, such as a concomitant effort to enhance detection and enforcement of corrupt activities or change the institutional culture away from bribe-taking, may actually act as reinforcing feedback that amplifies corruption. In the case of a health system, implementation of strategies targeted only one aspect of the system may not only by exacerbate corruption, but also by direct significant resources to a solution that is ultimately ineffective at achieving the intended goal (Figure 3C).

## Discussion

### Applying systems-thinking tools to address health sector corruption

As previously discussed, health systems are comprised of complex interactions between numerous actors. These systems are extremely heterogeneous in terms of structure, funding, incentives, resource allocation, etc. Furthermore, there are key differences in the socio-economic and political environments within LMICs and HICs that impact health systems within these countries, including the role of donors and development aid. Consequently, adapting an anti-corruption strategy that was developed in HICs to a health system in LMICs may do little to improve the system or result in unintended consequences that exacerbate corruption or further weaken the health system. These challenges of adaptation are highlighted by the aforementioned example of implementing an anti-corruption agency. For these reasons, corruption in the health sector, specifically within LMICs, is a problem in need of a systems-thinking approach.

Systems thinking has been previously applied to understand corruption in LMICs outside of the health sector (78, 79). These previously employed strategies can be combined with a health system strengthening framework put forth by de Savigny et al. (80) to better understand and disrupt health sector corruption. We propose a 4-part process to apply systems thinking to health sector corruption: qualitative analysis, developing a system map, designing an intervention, and developing an evaluation framework.

### Qualitative analysis

A qualitative analysis is an essential first step to a complete understanding of health sector corruption. Some have argued that corruption is particularly intractable because it serves a function in the system (78). Consequently, interventions that disrupt this function will be met with resistance. Based on studies cited above, the function of practices such as absenteeism, theft/diversion, and informal payments within the health sector of LMICs may include access to faster services or supplementing low salaries. However, given the heterogeneity of health systems globally, a local understanding is required to fully appreciate the role corruption plays in a given system.

A key component to this local understanding is getting input from actors at all levels of health sector, including those in positions of leadership (Figure 1). This qualitative input should focus on the informants' perceptions of, personal experiences with, and motivations underlying corruption in the health sector. Informants should also be asked about their impression of the health system more broadly, including their understanding of the incentives, configuration of leadership, regulations, renumeration structures, accountability structures, etc.

Analysis of this qualitative data can then be organized into themes that provide stakeholders with a better understanding of health sector corruption. As an example, qualitative analysis was performed by Scharbatke-Church et al. (81) to better understand corruption within the criminal justice system in Northern Uganda. Through this analysis, they identified several functions of corruption, including access to police or judges, maintaining power, or to generate revenue for operating costs to the maintain the system. Applying a similar strategy to the health sector has the potential to not only reveal to types of corruption that are occurring and the actors involved, but more importantly, its functions and the key dynamic relationships that enable corruption and maintain its role in the health system. Moreover, this deep understanding of the system will prevent inappropriate adaptation of anti-corruption programs that were utilized elsewhere.

### System mapping

The understanding of the system gained from the qualitative analysis can then be used to develop a causal loop diagram. The goal of the causal loop diagram is to visually represent the complex relationships between variables within the system that contribute to corruption (81). This approach was used in in Pakistan where Ullah et al. (79) conducted a thorough qualitative analysis focusing on citizens' experience with, perceptions of, and strategies for combatting corruption. Based on the themes extracted from this analysis, they created a comprehensive causal loop diagram modeling corruption in Pakistan that was inclusive of social, economic, legal, and political relationships. Through this process the authors identified several variables contributing to corruption that were under recognized in literature, such as the role of inflation, religious values, the size of government, and transparency in development aid. In Northern Uganda, a system map of the criminal justice system was essential to identifying both the drivers and enablers of corruption and the function that corruption serves in the system. This information was critical because most of the existing anti-corruption strategies in this region were only addressing enablers, not drivers, of corruption (78).

In the setting of health sector corruption, variables contributing to corruption may include suboptimal work conditions; low salaries for public sector workers; long wait times for services; scarcity of medications and/or medical supplies; lack of monitoring and accountability of health care workers, industry, suppliers, donor agencies, and policy makers; knowledge asymmetry between actors; corrupt behavior modeled by those in leadership positions; etc. After all the variables have been identified, one can use causal links to illustrate the dynamic relationships between variables. This system map complete with variables and causal links can help stakeholders identify reinforcing loops that exacerbate corruption or stabilizing loops that promote an equilibrium of corrupt behavior that becomes institutionalized within the health system. A potential example of how corruption can become institutionalized is the experience of public health care workers in rural Uganda who negotiated changes to facility workflow in order to accommodate for baseline staffing shortages due to pervasive absenteeism (53).

Furthermore, an understanding of these dynamic relationships is critical to anticipate temporal delays between and downstream effects of a precipitating factor and the ultimate outcome. Combatting corruption in the health sector is a long-term endeavor, understanding where delayed results could occur will prevent stakeholders or funders from prematurely abandoning an effective strategy where evidence of success may not be readily apparent. This comprehensive representation of the system is essential to designing an effective intervention.

### Designing (and refining) an intervention

After a health system and the impact of corruption on the system has been sufficiently mapped, an intervention can be developed. Using the format proposed by de Savigny et al. (80)designing an intervention starts with getting input from key stakeholders who represent different levels of the system and are positioned to understand areas that need to be improved. In the case of reducing health sector corruption in LMICs, these key stakeholders may include government officials and other policy-makers, donors, development organizations, payers, suppliers, providers, and patients. An ideal intervention should utilize a combination of measures that address different variables within the system (82). As highlighted by the anti-corruption strategies mentioned in previous sections, targeting one component of the system is unlikely to bring sustainable change. For instance, only addressing incentive structures by raising salaries without a concomitant effort to bolster monitoring and enforcement may perpetuate and even exacerbate corruption as seen in the example from Ghana (77).

Any potential intervention should then be applied to the system map to assess its effect on existing feedback loops, anticipate unintended consequences, and identify delayed outcomes. System dynamics modeling is one approach to this assessment. System dynamics modeling is an iterative process that utilizes mathematical modeling to predict the impact of various hypothetical scenarios on a given system (83). Information from these models can be used to further refine the intervention to mitigate negative downstream effects or unintended consequences.

### Developing an evaluation framework

Once an intervention has been designed and refined based on the system map, then an evaluation framework can be developed. However, there are some important features of corruption that must be considered when creating an evaluation strategy. First, the illicit nature of corruption makes it challenging to identify indicators of progress that can be reliably measured (84). Moreover, there is no clearly defined “road map” for successfully mitigating corruption in the health sector (64) and therefore typical monitoring and evaluation approaches for public health programs may not apply in this setting. Lastly, it will be challenging to anticipate every potential impact an intervention may have on systems as dynamic and resistant to change as health sector corruption in LMICs. For these reasons, evaluating the progress of anti-corruption strategies requires a non-traditional approach.

An example of such an approach has been previously described for a collective action intervention to reduce corruption in the criminal justice system of the DRC (84). Although a thorough systems analysis was performed at the outset, the authors describe a frequent monitoring and evaluation process characterized by an openness to challenge this initial analysis and make changes based on feedback collected after implementation of the intervention. Importantly, this feedback came from program participants rather than implementers (84). This example demonstrates that an iterative evaluation framework based on feedback from patients, providers, suppliers, and policy-makers may be preferable to a rigid evaluation plan with pre-defined indicators for success for addressing health sector corruption. In addition, frequent evaluation in the context of the system map should be included to make any changes to the intervention if necessary.

## Conclusion

Health care delivery results from an intricate series of interactions between numerous different actors within the system. It is clear that pervasive corruption is a detriment to effective health care delivery, particularly in LMICs. Addressing health sector corruption has the potential to strengthen health systems where they have historically been weak. However, due to the complexity and heterogeneity of health systems globally, a comprehensive understanding of the system structures that underly the individual instances and patterns of corrupt behavior is essential to developing an effective anti-corruption strategy. Anti-corruption strategies developed without this understanding are unlikely to result in meaningful improvements and may even further weaken health systems. Consequently, health sector corruption in LMICs is a problem in need of a system-thinking approach in order develop and successfully implement mitigation strategies that result in sustainable improvements in health systems and consequently, the health of populations.

## Author contributions

EG performed the literature review, applied the systems-thinking conceptual framework to corruption, and wrote the article.

## Conflict of interest

The author declares that the research was conducted in the absence of any commercial or financial relationships that could be construed as a potential conflict of interest.

## Publisher's note

All claims expressed in this article are solely those of the authors and do not necessarily represent those of their affiliated organizations, or those of the publisher, the editors and the reviewers. Any product that may be evaluated in this article, or claim that may be made by its manufacturer, is not guaranteed or endorsed by the publisher.

## References

[1] Transparency International - What is Corruption?. Available online at: https://www.transparency.org/what-is-corruption (accessed August 18, 2019).

[2] Factor R Kang M. Corruption and population health outcomes: an analysis of data from 133 countries using structural equation modeling. Int J Public Health. (2015) 60:633–41. 10.1007/s00038-015-0687-6 25994589

[3] Williams CC Horodnic AV. Rethinking informal payments by patients in Europe: an institutional approach. Health Policy Amst Neth. (2017) 121:1053–62. 10.1016/j.healthpol.2017.08.007 28867153

[4] Hanf M Van-Melle A Fraisse F Roger A Carme B Nacher M. Corruption kills: estimating the global impact of corruption on children deaths. PLoS ONE. (2011) 6:e26990. 10.1371/journal.pone.0026990 22073233 PMC3206868

[5] Lio MC Lee MH. Corruption costs lives: a cross-country study using an IV approach. Int J Health Plann Manage. (2016) 31:175–90. 10.1002/hpm.2305 26122874

[6] Li Q An L Xu J Baliamoune-Lutz M. Corruption costs lives: evidence from a cross-country study. Eur J Health Econ HEPAC Health Econ Prev Care. (2018) 19:153–65. 10.1007/s10198-017-0872-z 28197784

[7] Collignon P Athukorala PC Senanayake S Khan F. Antimicrobial resistance: the major contribution of poor governance and corruption to this growing problem. PLoS ONE. (2015) 10:e0116746. 10.1371/journal.pone.0116746 25786027 PMC4364737

[8] Nikoloski Z Mossialos E. Corruption, inequality and population perception of healthcare quality in Europe. BMC Health Serv Res. (2013) 13:472. 10.1186/1472-6963-13-472 24215401 PMC3831823

[9] Witvliet MI Kunst AE Arah OA Stronks K. Sick regimes and sick people: a multilevel investigation of the population health consequences of perceived national corruption. Trop Med Int Health TM IH. (2013) 18:1240–7. 10.1111/tmi.12177 24016030

[10] U.S. Department of Justice, U.S. Department of Health and Human Services. Health Care Fraud and Abuse Control Program Annual Report for Fiscal Year 2019. Washington, DC: U.S. Department of Health and Human Services Office of the Inspector General (2020).

[11] Shrank WH Rogstad TL Parekh N. Waste in the US health care system: estimated costs and potential for savings. JAMA. (2019) 322:1501–9. 10.1001/jama.2019.13978 31589283

[12] The Ignored Pandemic [Internet]. Transparency International. (2019). Available online at: http://ti-health.org/content/the-ignored-pandemic/ (accessed January 20, 2020)

[13] Habibov N Cheung A. Revisiting informal payments in 29 transitional countries: the scale and socio-economic correlates. Soc Sci Med. (2017) 178:28–37. 10.1016/j.socscimed.2017.02.003 28192744

[14] Horodnic AV Mazilu S Oprea L. Drivers behind widespread informal payments in the Romanian public health care system: from tolerance to corruption to socio-economic and spatial patterns. Int J Health Plann Manage. (2018) 33:e597–611. 10.1002/hpm.2509 29542181

[15] Hsiao A Vogt V Quentin W. Effect of corruption on perceived difficulties in healthcare access in sub-Saharan Africa. PLoS ONE. (2019) 14:e0220583. 10.1371/journal.pone.0220583 31433821 PMC6703670

[16] Nicholas LH Segal J Hanson C Zhang K Eisenberg MD. Medicare beneficiaries' exposure to fraud and abuse perpetrators. Health Aff Proj Hope. (2019) 38:788–93. 10.1377/hlthaff.2018.05149 31059371

[17] Nicholas LH Hanson C Segal JB Eisenberg MD. Association between treatment by fraud and abuse perpetrators and health outcomes among medicare beneficiaries. JAMA Intern Med. (2020) 180:62–9. 10.1001/jamainternmed.2019.4771 31657838 PMC6820041

[18] TI Publication - Global Corruption Report 2006: Corruption and health. Available online at: https://www.transparency.org/whatwedo/publication/global_corruption_report_2006_corruption_and_health (accessed July 08, 2022).

[19] Wouters OJ. Lobbying Expenditures and Campaign Contributions by the Pharmaceutical and Health Product Industry in the United States, 1999–2018. JAMA Intern Med. (2018) 180:688–97. 10.1001/jamainternmed.2020.0146 32125357 PMC7054854

[20] Couffinhal A Frankowski A. Wasting with intention: Fraud, abuse, corruption and other integrity violations in the health sector. In: Tackling Wasteful Spending on Health. Paris: OECD Publishing (2017). p. 265–301. Available online at: https://www.oecd-ilibrary.org/social-issues-migration-health/tackling-wasteful-spending-on-health/wasting-with-intention-fraud-abuse-corruption-and-other-integrity-violations-in-the-health-sector_9789264266414-10-en

[21] Mitchell JM. Urologists' self-referral for pathology of biopsy specimens linked to increased use and lower prostate cancer detection. Health Aff Proj Hope. (2012) 31:741–9. 10.1377/hlthaff.2011.1372 22492891

[22] Nguyen TA Knight R Mant A Razee H Brooks G Dang TH. Corruption practices in drug prescribing in Vietnam - an analysis based on qualitative interviews. BMC Health Serv Res. (2018) 18:587. 10.1186/s12913-018-3384-3 30055601 PMC6064099

[23] Jürges H Köberlein J. What explains DRG upcoding in neonatology? The roles of financial incentives and infant health. J Health Econ. (2015) 43:13–26. 10.1016/j.jhealeco.2015.06.001 26114589

[24] King K. Medicare Fraud: Progress Made, but More Action Needed to Address Medicare Fraud, Waste, and Abuse. United States Government Accountability Office (2014). Available online at: https://www.gao.gov/products/gao-14-560t (accessed May 06, 2022).

[25] AP: Fraud Plagues Global Health Fund. CBS News [Internet]. Available online at: https://www.cbsnews.com/news/ap-fraud-plagues-global-health-fund/ (accessed July 8, 2022).

[26] Ferrinho P Omar MC Fernandes MDJ Blaise P Bugalho AM Lerberghe WV. Pilfering for survival: how health workers use access to drugs as a coping strategy. Hum Resour Health. (2004) 2:4. 10.1186/1478-4491-2-4 15115548 PMC411059

[27] Lindelow M Serneels P. The performance of health workers in Ethiopia: results from qualitative research. Soc Sci Med. (2006) 62:2225–35. 10.1016/j.socscimed.2005.10.015 16309805

[28] Maini R Hotchkiss DR Borghi JA. cross-sectional study of the income sources of primary care health workers in the Democratic Republic of Congo. Hum Resour Health. (2017) 15:17. 10.1186/s12960-017-0185-4 28219445 PMC5322790

[29] McPake B Asiimwe D Mwesigye F Ofumbi M Ortenblad L Streefland P. Informal economic activities of public health workers in Uganda: implications for quality and accessibility of care. Soc Sci Med. (1982) 49:849–65. 10.1016/S0277-9536(99)00144-6 10468391

[30] Ackers L Ioannou E Ackers-Johnson J. The impact of delays on maternal and neonatal outcomes in Ugandan public health facilities: the role of absenteeism. Health Policy Plan. (2016) 31:1152–61. 10.1093/heapol/czw046 27142803 PMC5035777

[31] Belita A Mbindyo P English M. Absenteeism amongst health workers–developing a typology to support empiric work in low-income countries and characterizing reported associations. Hum Resour Health. (2013) 11:34. 10.1186/1478-4491-11-34 23866770 PMC3721994

[32] Bouchard M Kohler JC Orbinski J Howard A. Corruption in the health care sector: a barrier to access of orthopaedic care and medical devices in Uganda. BMC Int Health Hum Rights. (2012) 12:5. 10.1186/1472-698X-12-5 22554349 PMC3492067

[33] Chaudhury N Hammer J Kremer M Muralidharan K Rogers FH. Missing in action: teacher and health worker absence in developing countries. J Econ Perspect J Am Econ Assoc. (2006) 20:91–116. 10.1257/089533006776526058 17162836

[34] Ferrinho P Van Lerberghe W Julien MR Fresta E Gomes A Dias F. How and why public sector doctors engage in private practice in Portuguese-speaking African countries. Health Policy Plan. (1998) 13:332–8. 10.1093/heapol/13.3.332 10187602

[35] Gruen R Anwar R Begum T Killingsworth JR Normand C. Dual job holding practitioners in Bangladesh: an exploration. Soc Sci Med. (1982) 54:267–79. 10.1016/S0277-9536(01)00026-0 11824931

[36] Jan S Bian Y Jumpa M Meng Q Nyazema N Prakongsai P. Dual job holding by public sector health professionals in highly resource-constrained settings: problem or solution? Bull World Health Organ. (2005) 83:771–6. 16283054 PMC2626421

[37] Onwujekwe O Agwu P Orjiakor C McKee M Hutchinson E Mbachu C. Corruption in Anglophone West Africa health systems: a systematic review of its different variants and the factors that sustain them. Health Policy Plan. (2019) 5:70. 10.1093/heapol/czz070 31377775 PMC6788210

[38] Tweheyo R Daker-White G Reed C Davies L Kiwanuka S Campbell S. “Nobody is after you; it is your initiative to start work”: a qualitative study of health workforce absenteeism in rural Uganda. BMJ Glob Health. (2017) 2:e000455. 10.1136/bmjgh-2017-000455 29527333 PMC5841506

[39] Agwu P Ogbozor P Odii A Orjiakor C Onwujekwe O. Private money-making indulgence and inefficiency of primary healthcare in Nigeria: a qualitative study of health workers' absenteeism. Int J Public Health. (2020) 65:1019–26. 10.1007/s00038-020-01405-3 32840632 PMC7497334

[40] McPake B Russo G Hipgrave D Hort K Campbell J. Implications of dual practice for universal health coverage. Bull World Health Organ. (2016) 94:142–6. 10.2471/BLT.14.151894 26908963 PMC4750430

[41] Lewis M. Informal payments and the financing of health care in developing and transition countries. Health Aff Proj Hope. (2007) 26:984–97. 10.1377/hlthaff.26.4.984 17630441

[42] Cherecheş RM Ungureanu MI Sandu P Rus IA. Defining informal payments in healthcare: a systematic review. Health Policy Amst Neth. (2013) 110:105–14. 10.1016/j.healthpol.2013.01.010 23410757

[43] Akwataghibe N Samaranayake D Lemiere C Dieleman M. Assessing health workers' revenues and coping strategies in Nigeria–a mixed-methods study. BMC Health Serv Res. (2013) 13:387. 10.1186/1472-6963-13-387 24093219 PMC3853328

[44] Schaaf M Topp SM. A critical interpretive synthesis of informal payments in maternal health care. Health Policy Plan. (2019) 34:216–29. 10.1093/heapol/czz003 30903167 PMC6528746

[45] Stringhini S Thomas S Bidwell P Mtui T Mwisongo A. Understanding informal payments in health care: motivation of health workers in Tanzania. Hum Resour Health. (2009) 7:53. 10.1186/1478-4491-7-53 19566926 PMC2711965

[46] Vian T Gryboski K Grybosk K Sinoimeri Z Hall R. Informal payments in government health facilities in Albania: results of a qualitative study. Soc Sci Med. (2006) 62:877–87. 10.1016/j.socscimed.2005.07.005 16115713

[47] World Health Organization. WHO global surveillance and monitoring system for substandard and falsified medical products [Internet]. World Health Organization. (2017). 64 p. Available online at: https://apps.who.int/iris/handle/10665/326708 (accessed July 8, 2022).

[48] Bate R Mathur A. Corruption and medicine quality in Latin America: A Pilot Study. BE J Econ Anal Policy. (2018) 18. Available online at: https://www.degruyter.com/view/j/bejeap.2018.18.issue-2/bejeap-2017-0076/bejeap-2017-0076.xml?lang=en 10.1515/bejeap-2017-0076 (accessed January 22, 2022).

[49] Corruption Perceptions Index 2021. Transparency International (2022). Available online at: https://www.transparency.org/en/cpi/2021 (accessed June 22, 2022).

[50] World Bank Country and Lending Groups – World Bank Data Help Desk. Available online at: https://datahelpdesk.worldbank.org/knowledgebase/articles/906519-world-bank-country-and-lending-groups (accessed April 4, 2022).

[51] Persson A Rothstein B Teorell J. Rethinking the nature of the grabbing hand. In: Holmberg S Rothstein B editors. Good Government: The Relevance of Political Science. Edward Elgar Publishing (2014). p. 251–73.

[52] Stroh DP. Systems Thinking for Social Change: A Practical Guide to Solving Complex Problems, Avoiding Unintended Consequences, And Achieving Lasting Results. White River Junction, Vermont: Chelsea Green Publishing (2015).

[53] Tweheyo R Reed C Campbell S Davies L Daker-White G. “I have no love for such people, because they leave us to suffer”: a qualitative study of health workers' responses and institutional adaptations to absenteeism in rural Uganda. BMJ Glob Health. (2019) 4:e001376. 10.1136/bmjgh-2018-001376 31263582 PMC6570979

[54] Bauhr M. Need vs. greed corruption. In: Holmberg S Rothstein B editors. Good Government: The Relevance of Political Science. Cheltenham: Edward Elgar Publishing (2014). p. 68–86.

[55] Kruk ME Gage AD Arsenault C Jordan K Leslie HH Roder-DeWan S. High-quality health systems in the sustainable development goals era: time for a revolution. Lancet Glob Health. (2018) 6:e1196–252. 10.1016/S2214-109X(18)30386-3 30196093 PMC7734391

[56] Pfeiffer J Chapman R. Anthropological perspectives on structural adjustment and public health. Annu Rev Anthropol. (2010) 39:149–65. 10.1146/annurev.anthro.012809.1051012401150

[57] Moss T. African Development: Making Sense of the Issues and Actors. Third edition Boulder, Colorado: Lynne Rienner Publishers, Inc. (2018).

[58] Persson A Rothstein B Teorell J. Why anticorruption reforms fail—Systemic corruption as a collective action problem. Governance. (2013) 26:449–71. 10.1111/j.1468-0491.2012.01604.x

[59] Dieleman JL Graves C Johnson E Templin T Birger M Hamavid H. Sources and focus of health development assistance, 1990–2014. JAMA. (2015) 313:2359–68. 10.1001/jama.2015.5825 26080340

[60] Alesina A Weder B. Do corrupt governments receive less foreign aid? Am Econ Rev. (2002) 92:1126–37. 10.1257/00028280260344669

[61] Easterly W Williamson CR. Rhetoric vs. reality: the best and worst of aid agency practices. World Dev. 2011 Nov 1;39(11):1930–49. 10.1016/j.worlddev.2011.07.027

[62] Bräutigam DA Knack S. Foreign aid, institutions, and governance in Sub-Saharan Africa. Econ Dev Cult Change. (2004) 52:255–85. 10.1086/38059228704674

[63] Kohler JC Bowra A. Exploring anti-corruption, transparency, and accountability in the world health organization, the united nations development programme, the world bank group, and the global fund to fight AIDS, tuberculosis and Malaria. Glob Health. (2020) 16:101. 10.1186/s12992-020-00629-5 33081805 PMC7573869

[64] Gaitonde R Oxman AD Okebukola PO Rada G. Interventions to reduce corruption in the health sector. Cochrane Database Syst Rev. (2016) (8):CD008856. 10.1002/14651858.CD008856.pub2 27528494 PMC5014759

[65] Huss R Green A Sudarshan H Karpagam S Ramani K Tomson G. Good governance and corruption in the health sector: lessons from the Karnataka experience. Health Policy Plan. (2011) 26:471–84. 10.1093/heapol/czq080 21169338

[66] Peiffer C Armytage R Marquette H. “Islands of integrity”? Reductions in bribery in Uganda and South Africa and lessons for anti-corruption policy and practice. (2018). Available online at: https://www.dlprog.org/publications/research-papers/islands-of-integrity-reductions-in-bribery-in-uganda-and-south-africa-and-lessons-for-anti-corruption-policy-and-practice (accessed March 17, 2022).

[67] Di Tella R Savedoff WD. Diagnosis Corruption Fraud in Latin America's Public Hospitals. Washington DC: Inter-American Development Bank (2001). 226 p.

[68] Bjorkman Nyqvist M de Walque D Svensson J. Information is Power: Experimental Evidence on the Long-Run Impact of Community Based Monitoring. Washington, DC: World Bank (2014). Available online at: https://openknowledge.worldbank.org/handle/10986/20364 10.1596/1813-9450-7015 (accessed March 15, 2022).

[69] Arosteguí J Hernandez C Suazo H Cárcamo A Reyes RM Andersson N. Auditing Nicaragua's anti-corruption struggle, 1998 to 2009. BMC Health Serv Res. (2011) 11(Suppl 2):S3. 10.1186/1472-6963-11-S2-S3 22375610 PMC3332562

[70] Arkedis J Creighton J Dixit A Fung A Kosack S Levy D. Can Transparency and Accountability Programs Improve Health? Experimental Evidence from Indonesia and Tanzania [Internet]. Harvard Kennedy School. (2019). (HKS Faculty Research Working Paper Series). Report No: RWP19-020. Available online at: https://www.hks.harvard.edu/publications/can-transparency-and-accountability-programs-improve-health-experimental-evidence 10.2139/ssrn.3399124 (accessed March 1, 2022).

[71] Cheeseman N Peiffer C. The unintended consequences of anti-corruption messaging in Nigeria: Why pessimists are always disappointed. Anti-Corruption Evidence. (2020). Report No. 024. Available online at: https://ace.soas.ac.uk/publication/unintended-consequences-of-anti-corruption-messaging-in-nigeria/ (accessed March 1, 2022).

[72] Corbacho A Gingerich DW Oliveros V Ruiz-Vega M. Corruption as a self-fulfilling prophecy: evidence from a survey experiment in Costa Rica. Am J Polit Sci. (2016) 60:1077–92. 10.1111/ajps.12244

[73] Global Burden of Disease Health Financing Collaborator Network. Past, present, and future of global health financing: a review of development assistance, government, out-of-pocket, and other private spending on health for 195 countries, 1995–2050. Lancet Lond Engl. (2019) 393:2233–60. 10.1016/S0140-6736(19)30841-4 31030984 PMC6548764

[74] National National Academies of Sciences Engineering and Medicine Health and Medicine Division Board Board on Health Care Services Board Board on Global Health Committee Committee on Improving the Quality of Health Care Globally. Crossing the Global Quality Chasm: Improving Health Care Worldwide [Internet]. Washington (DC): National Academies Press (US) (2018). (The National Academies Collection: Reports funded by National Institutes of Health). Available online at: http://www.ncbi.nlm.nih.gov/books/NBK535653/ (accessed January 20, 2022).

[75] Le VH de Haan J Dietzenbacher E. Do Higher Government Wages Reduce Corruption? Evidence Based on a Novel Dataset. Rochester, NY: Social Science Research Network (2013). Report No. ID 2274648. Available online at: https://papers.ssrn.com/abstract=2274648 (accessed March 8, 2022).

[76] Van Rijckeghem C Weder B. Bureaucratic corruption and the rate of temptation: do wages in the civil service affect corruption, and by how much? J Dev Econ. (2001) 65:307–31. 10.1016/S0304-3878(01)00139-0

[77] Foltz JD Opoku-Agyemang KA. Do higher salaries lower petty corruption? A policy experiment on West Africa's highways [Internet]. International Growth Centre. (2015). Available online at: https://www.gov.uk/research-for-development-outputs/do-higher-salaries-lower-petty-corruption-a-policy-experiment-on-west-africa-s-highways (accessed March 1, 2022).

[78] Scharbatke-Church C Chigas DV. Using systems thinking to understand and address corruption in the criminal justice system of fragile state. In: Corruption, Social Sciences and the Law: Exploration Across the Disciplines. London, New York: Routledge (2019). p. 201–22. 10.4324/9780429197352-12

[79] Ullah MA Urquhart C Arthanari T Ahmed E. Dimensions of corruption in Pakistan: a systems thinking approach and qualitative analysis. Syst Res Behav Sci. (2022) 39:324–38. 10.1002/sres.2775

[80] De Savigny D Taghreed A. Systems thinking for health systems strengthening. Alliance for Health Policy and Systems Research. World Health Organization (2009). Available online at: https://apps.who.int/iris/handle/10665/44204 (accessed June 13, 2022).

[81] Scharbatke-Church C Chigas DV. Facilitation in the Criminal Justice System [Internet]. Medford, MA: Institute for Human Security, The Fletcher School, Tufts University (2016). Report No. Series 1, Number 2. Available online at: https://sites.tufts.edu/ihs/facilitation-in-the-criminal-justice-system/ (accessed June 22, 2022).

[82] Hussman K. Health sector corruption: Practical recommendations for donors [Internet]. U4 Anti-Corruption Resource Center (2020). Available online at: https://www.u4.no/publications/health-sector-corruption (accessed June 2, 2022).

[83] Homer JB Hirsch GB. System dynamics modeling for public health: background and opportunities. Am J Public Health. (2006) 96:452–8. 10.2105/AJPH.2005.062059 16449591 PMC1470525

[84] Scharbatke-Church C Barnard-Webster K Woodrow P. Collective Action Against Corruption in the Criminal Justice System [Internet]. Cambridge, MA: CDA Collaborative Learning Projects (2017). Available online at: https://www.cdacollaborative.org/publication/collective-action-corruption-criminal-justice-system/ (accessed June 22, 2022).

